# Antenatal care utilization during the COVID-19 pandemic: an online cross-sectional survey among Filipino women

**DOI:** 10.1186/s12884-022-05234-5

**Published:** 2022-12-12

**Authors:** Glaiza S. de Guzman, Maria Jesusa B. Banal-Silao

**Affiliations:** Department of Obstetrics and Gynecology, University of the Philippines Manila – Philippine General Hospital, Manila, Philippines

**Keywords:** Antenatal care, COVID-19, Philippines, Pandemic

## Abstract

**Background:**

The COVID-19 pandemic resulted in unprecedented challenges to healthcare systems worldwide, including interruption of antenatal care services. The study aimed to determine the utilization of antenatal care services of Filipino women during the COVID-19 pandemic.

**Methods:**

A cross-sectional study was conducted among postpartum women using an online self-administered survey in the Philippines from January 1 to March 31, 2022. The questionnaire used to assess health-seeking behavior was validated before the survey proper. Women aged 18 to 45 years who delivered in 2021 were recruited. The participants answered a structured questionnaire to assess their access, perceptions, and utilization of antenatal care. Utilization of antenatal care was evaluated using standard measures, including the timing of initiation of antenatal care, number of subsequent visits, and place of consults. The factors affecting the adequacy of antenatal care were determined for each variable through simple logistic regression.

**Results:**

A total of 318 women were enrolled in the study. All the respondents agreed on the necessity of antenatal care. However, only 46.37% had six or more in-person antenatal visits, with the majority attended to by midwives at community health centers. Most respondents (71.38%) initiated antenatal care during the first trimester. Almost half reported deferrals of visits mainly due to lockdown restrictions, transportation problems, and financial issues. Positive predictors of adequate antenatal care were prior pregnancies (OR 1.80, 95% CI 1.11–9.20 for 2–3 prior pregnancies; OR 3.02, 95% CI 1.45–6.29 for 4 or more prior pregnancies), live births (OR 1.67, 95% CI 1.04–2.69 for 2–3 prior live births; OR 2.46, 95% CI 1.17–5.16 for 4 or more prior live births), having living children (OR 1.74, 95% CI 1.09–2.79), spousal support (OR 1.75, 95% CI 1.01–3.03 for married women; OR 1.89, 95% CI 1.09–3.28 for women with common-law partners), history of obstetric complications (OR 2.82, 95% CI 1.33–5.97), and use of private vehicles (OR 2.65, 95% CI 1.05–6.68). Negative predictors were employment (OR 0.37, 95% CI 0.22–0.63) and medical examination prior to pregnancy (OR 0.36, 95% CI 0.23–0.58).

**Conclusion:**

Despite an overall positive perception of the necessity of antenatal care, utilization has been inadequate in more than half of the respondents. Various individual, facility, and policy-level factors affected the utilization of services during the pandemic. There is a need to augment antenatal care services in the country by mitigating barriers to access. The public health response should strengthen collaborative efforts with primary-level healthcare to increase service provision, especially to more vulnerable populations.

## Background

A pandemic state for COVID-19 was declared by the World Health Organization last March 11, 2020 [1]. The rapid spread and evolution of the infection have dramatically changed medical practice worldwide. It affected healthcare delivery and continued to have disproportionate effects on reproductive health, including the provision of antenatal care services. Healthcare systems were reorganized to divert personnel and resources to the pandemic response.

Most countries redesigned antenatal care services to decrease the exposure of pregnant women to infected individuals. The number of routine antenatal care visits was reduced to six in-person and two telemedicine consults [2]. The Philippine Obstetrical and Gynecological Society (POGS) with the Philippine Society of Maternal-Fetal Medicine (PSMFM) [3] adopted similar recommendations of six scheduled visits and telemedicine consults as needed. Pregnant women were encouraged to observe and maintain antenatal care appointments. Initiation of consult through a telemedicine platform was suggested for women at less than 11 weeks of gestation. The number of actual antenatal care visits was reduced by timing the visits to include indicated laboratory tests and fetal wellbeing studies. At least six antenatal visits were suggested at 11–13 weeks, 20 weeks, 28 weeks, 32 weeks, 36 weeks, and 37 weeks to delivery. Laboratory tests were encouraged in lower-load facilities to minimize patient exposure. Consults between these periods were encouraged to be carried out through voice and video consults. These adaptations allowed facilities to allocate limited slots for in-person consults to high-risk patients who require more visits and close monitoring.

A woman’s decision to seek antenatal care is affected by various factors, including personal needs and circumstances, sociodemographic characteristics, and access to health care [4]. It is essential to acknowledge that pregnant women may avoid in-person consultations for safety concerns and transportation problems. Women may also have difficulties adhering to antenatal care visits because they need to assist with the remote education of their older children and work from home [1].

Before this study, there was limited evidence on the utilization and adequacy of antenatal care in the Philippines during the COVID-19 pandemic. The current study sought to evaluate the factors affecting the health-seeking behavior of Filipino women for obstetric care, identify the health-seeking patterns for antenatal visits, and determine the adequacy of consults during the COVID-19 pandemic.

### Operative definition of terms

The study evaluated the utilization of antenatal care by Filipino women. Adequate antenatal care in this study was measured using the following variables:

1. Sufficiency: Defined as having at least six in-person antenatal consults recommended by the Philippine Obstetrical and Gynecological Society with the Philippine Society of Maternal-Fetal Medicine [3].
2. Timeliness: Defined as the initiation of antenatal care during the first trimester.
3. Antenatal care provided by a skilled health attendant, including doctors, nurses, or midwives.

The study did not evaluate the adequacy of antenatal care in terms of appropriateness in content including the procedures and processes during antenatal care. The adequacy was determined using sufficiency, timeliness, and provision by a skilled attendant.

## Methods

### Study design and sample size

A cross-sectional observational study was conducted among postpartum Filipino women from January 1, 2022 to March 31, 2022 using an online self-administered questionnaire. The sample size was calculated at 318 based on the report of Tadesse et al. [5] that the proportion of women who completed antenatal care in facilities is 29.3%. This was calculated using Epi Info with a margin of error of 5% and a confidence level of 95%. To ensure the adequacy of the sample size for factor analysis, the value of OR 0.61 was used to assess the effect of distance on ANC utilization [6].

### Questionnaire development and validation

The items on the questionnaire were adapted from a study among pregnant women in China by Liu et al. [6]. The questionnaire by Liu et al. contained items categorized into environmental factors and population characteristics affecting health-seeking behavior. Modifications were made to include the geographic region of the participants. Item on the distance to the nearest facility was amended to indicate the participant’s travel time. Questions on the marital status, employment status, number of living children, timing of initiation of antenatal visit, number and type of consults, providers of antenatal care, type of institution for delivery were added. Lastly, an item on whether the pandemic affected their choice of healthcare facility was included.

Four obstetrician-gynecologists reviewed the items on the questionnaire. The experts indicated their decision to remove, keep, or modify each item. All items were kept except for health insurance schemes. Women about to give birth in the Philippines are enrolled to the national insurance at point of care. Choices for some questions were modified to the Philippine setting (i.e., types of facilities and transportation). The questionnaire was translated into Filipino and back-translated into English. A panel of ten obstetrician-gynecologists reviewed the translated questionnaire to check the appropriateness of the forward translated questions. The panel evaluated the level of relevance of each item on a four-point scale (1 = not relevant, 2 = somewhat relevant, 3 = quite relevant, 4 = highly relevant).

Content and face validity testing was done with any suggestions or comments incorporated into the questionnaire. For validation, pilot testing was performed on a group similar to the study respondents. The content validity index for items (I-CVI) and scale-level content validity index universal agreement (S-CVI/UA) were computed to indicate the content validity. Twenty-one items had an I-CVI of 1.0. Only one item on whether pregnancy was planned had an I-CVI of 0.70 and was therefore removed. The remaining questions had I-CVIs of 0.80 to 0.90. The S-CVI/UA was 0.78, and the S-CVI Ave was 0.96. A total of 31 items were retained, and suggestions were incorporated into the questionnaire for pilot testing.

The questionnaire was divided into four parts, including items on the sociodemographic data, obstetric and medical history, access and perceptions of antenatal care, and actual utilization of antenatal care. Utilization of antenatal care was evaluated using standard measures, including the timing of initiation of antenatal care, number of subsequent visits, and place of consults. Face validity testing was done on ten patients. The questionnaire was modified according to their suggestions.

The questionnaire was administered to ten respondents and re-administered within one week. The intraclass correlation coefficient was computed to be 1.0 (95% CI: 0.99-1.00), signifying excellent reliability.

### Description of study procedures and study population

An invitation to participate in the study was advertised on different social media platforms and distributed to pregnancy-specific professional communities for distribution. Interested women were screened to determine their eligibility to participate. Eligible subjects were Filipino women aged 18 to 45 who delivered in 2021. Women younger than 18 years and those whose pregnancy resulted in a miscarriage were excluded from the study. Minors were excluded because parental or guardian consent cannot be obtained through the online survey. One of the study objectives was to evaluate the number of antenatal care visits during pregnancy to delivery and thus women whose pregnancies resulted in a miscarriage were excluded.

Selection bias was minimized by distributing the questionnaire to various online platforms to improve visibility. Obstetricians, residents-in-training, and midwives in both private and public health sectors were likewise encouraged to invite patients to the study. Interested participants referred to the study but had no internet access were aided by research assistants by phone call.

Interested participants were directed to an online form determining their eligibility to participate. Women who answered “Yes” to being Filipino, aged 18 to 45, and having given birth in 2021 were redirected to the survey proper. Meanwhile, having at least one “No” response identified interested participants as being ineligible and were not redirected to the questionnaire. Their participation eligibility was confirmed via a phone call verifying the data provided.

A user-friendly design and layout were used for the online questionnaire. Multiple responses to the online survey were avoided by restricting one response to a registered email address and by phone call verification after submission of the questionnaire. A pilot testing was performed to ensure clear and adequate instructions, the feasibility of the technology, ordering of the questions, and completeness of the contents.

Women who satisfied both the inclusion and exclusion criteria were redirected and invited to the survey proper. Only eligible women who completed the questionnaire were included in the study. The participants were allowed to choose the language of the survey. Consecutive enrollment of women was done until the sample size was met.

### Data analysis

Descriptive statistics were generated for all variables of interest. Mean with standard deviation and frequency with proportion were presented for quantitative and qualitative variables, respectively. The factors affecting the adequacy of antenatal care were determined through simple logistic regression. Variables tested included highest educational attainment, marital status, employment status, monthly household income, geographic location, number of past pregnancies, number of abortions, number of prior live births, number of living children, presence of concomitant medical diseases, history of pregnancy complications, place of birth of most recent pregnancy, distance to the nearest facility for antenatal care, mode of transportation, waiting time experienced, awareness of policies, participation in medical examination before pregnancy, and healthcare provider.

Odds ratios with 95% confidence intervals and *p*-values were presented to determine the statistically associated factors with the adequacy of utilization. All variables for analysis were screened for adequacy for logistic regression by ensuring that all categories have at least five (5) in cells. Cells with a frequency of less than five were merged with other cells to ensure adequacy for analysis. All analyses were done in STATA 17.0/BE, and *p*-values < 0.05 were considered statistically significant.

## Results

A total of 340 participants volunteered for the study, with 318 qualified respondents. All 318 eligible respondents completed the questionnaire and were enrolled in the study. The mean age of the respondents was 27.5 ± 5.5 years. Majority were high school graduates (40.25%), single (33.65%), unemployed (71.07%), and with a monthly household income of less than Php 20,000 (90.88%). The respondents were from the different geographic regions in the country, with nearly half (48.11%) from the National Capital Region. The sociodemographic characteristics of the study population are summarized in Table 1.

**Table 1:** Sociodemographic characteristics of the study population

Characteristic		N (*n* = 318)	Percentage
Highest educational attainment			
	Grade school	12	3.77%
	High school	128	40.25%
	College undergraduate	80	25.16%
	College graduate	74	23.27%
	Vocational course	15	4.72%
	Postgraduate course	9	2.83%
Marital status			
	Single	107	33.65%
	Married	105	33.02%
	Divorced/Separated	1	0.31%
	Common-law partner	105	33.02%
Employment status			
	Employed	92	28.93%
	Unemployed	226	71.07%
Monthly household income			
	Less than 20,000	289	90.88%
	20,000 to 50,000	24	7.55%
	More than 50,000	5	1.57%

Half of the respondents have had only one past pregnancy (46.54%). Majority had no miscarriages (85.22%) and did not report any pregnancy complications (88.68%). Of the 318 subjects, 298 (93.71%) did not have an existing medical illness. More than half (55.97%) gave birth in a hospital (Table 2).

It should be noted that nine women in the study delivered at home, going against the recommendation to deliver in facilities with skilled birth attendants capable of handling obstetric emergencies. Four of these women did not have antenatal visits and cited that the nearest healthcare facility was more than one hour of travel from their homes.

**Table 2:** Medical and obstetric histories of the study population

Characteristic		N (*n* = 318)	Percentage
Number of past pregnancies			
	1	148	46.54%
	2–3	131	41.20%
	4 or more	39	12.26%
Number of miscarriages			
	0	271	85.22%
	1	38	11.95%
	2 or more	9	2.83%
Number of deliveries or times given birth			
	1	160	50.31%
	2–3	122	38.36%
	4 or more	36	11.32%
Number of living children			
	0	1	0.31%
	1	159	50.00%
	2–3	129	40.57%
	4 or more	29	9.12%
Existing medical illness			
	With	20	6.29%
	Without	298	93.71%
History of pregnancy complications			
	With	36	11.32%
	Without	282	88.68%
Place of birth of most recent pregnancy			
	Home	9	2.83%
	Hospital	178	55.97%
	Lying-in clinic	131	41.19%

### Access to and perceptions of antenatal care

All the respondents agreed on the necessity of antenatal care. Most travel less than 30 min to the nearest facility and utilize public vehicles, as summarized in Table 3. More than half of the subjects wait for 15 to 30 min before being seen by their healthcare provider.

**Table 3:** Access to and perceptions of antenatal care

Characteristic		N (*n* = 318)	Percentage
Distance to the nearest facility for antenatal care			
	Less than 30 min	194	61.01%
	30 min to 1 h	92	28.93%
	More than 1 h	32	10.06%
Mode of transportation (allow to choose more than 1)			
	Private vehicle	22	6.92%
	Jeepney	93	29.25%
	Tricycle	159	50.00%
	Taxi	22	6.92%
	On foot	49	15.41%
	Motorcycle	52	16.35%
	Bus	7	2.20%
	MRT	8	2.52%
	FX	1	0.31%
	Bike	1	0.31%
Waiting time to see a healthcare provider			
	< 15 min	48	15.09%
	15–30 min	166	52.20%
	31–60 min	55	17.30%
	> 60 min	49	15.41%
Subjective feeling that antenatal advice applies to them			
	Yes	313	98.43%
	No	5	1.57%
Awareness of policies			
	Yes	265	83.33%
	No	53	16.67%
Participated in any health education before pregnancy			
	Yes	199	62.58%
	No	119	37.42%
Participated in medical examination before pregnancy			
	Yes	201	63.21%
	No	117	36.79%

Almost all respondents felt that the antenatal advice applied to their status. Most (83.33%) were aware of national policies on the provision of antenatal care. Majority have participated in health education (62.58%) and medical examinations (63.21%) before pregnancy.

### Utilization of antenatal care services

Regarding the timing of the first antenatal care visit, 71.38% were able to seek consult during the first trimester. Majority had more than six antenatal checkups (44.03%), primarily by face-to-face consult. Only 46.37% had adequate antenatal visits, defined by having six or more face-to-face checkups. Most respondents were attended to by midwives (50.31%) at community or barangay health centers (46.88%).

Almost half (45.28%) reported canceling antenatal visits. Among the cited reasons for deferrals were transportation problems (16.35%), lockdown or quarantine restrictions (16.04%), financial and employment status problems (12.58%), fear of going to the hospital (5.97%), full schedules of hospitals or clinics (5.35%), and lack of companion (6.92%).

The choice of health facility was affected by the pandemic in 75.47% of the subjects. Perception of the adequacy of antenatal care, which reflects the subjective feeling of the respondents of having received adequate care during pregnancy, was reported by 295 (92.77%) respondents. Meanwhile, the 23 respondents who perceived inadequacy named facility problems, healthcare providers in a hurry, unexpected poor pregnancy outcomes, and inability to afford antenatal care services. Data on the utilization of antenatal care are summarized in Table 4.

**Table 4:** Utilization of antenatal care services during the COVID-19 pandemic

Characteristic		N (*n* = 318)	Percentage
Timing of first antenatal visit			
	First trimester	227	71.38%
	Second trimester	72	22.64%
	Third trimester	15	4.72%
	No prenatal checkup	4	1.26%
Total number of antenatal checkups			
	0	4	1.26%
	1–6	174	54.71%
	More than 6	140	44.03%
Number of teleconsults			
	0	180	56.60%
	1–6	112	35.12%
	More than 6	26	8.18%
Number of face-to-face consults			
	0	14	4.42%
	1–6	176	55.52%
	More than 6	127	40.06%
Facility of face-to-face consults			
	Community or barangay health center	149	46.86
	Lying-in clinic	129	40.57%
	Public hospital	89	27.99%
	Private obstetrician	48	15.09%
	Rural Health Unit/City Health Office	2	0.63%
Healthcare provider			
	Doctor	158	49.69%
	Midwife	160	50.31%
	Nurse	89	27.99%
	Traditional Birth Attendant	11	3.46%

### Factors affecting adequacy of antenatal care

Married women and those with common-law partners were 1.75 and 1.89 times more likely to have adequate antenatal care than single women. Mothers who were employed have 63% less odds of having adequate prenatal checkups than those unemployed. Women who have had previous pregnancies (OR 1.80, CI 1.11–2.90 for 2 to 3 past pregnancies; OR 3.02, CI 1.45–6.29 for 4 or more past pregnancies ), livebirths (OR 1.67, CI 1.04–2.69 for 2 to 3 prior live births; OR 2.46, CI 1.17–5.16 for 4 or more prior live births), living children (OR 1.74, CI 1.09–2.79 for 2 to 3 living children) were more likely to have adequate consults (Table 5).

Similarly, mothers who had complications in their last pregnancy are 2.82 times more likely to have adequate prenatal visits than those who did not have complications. The use of private vehicles was associated with a 2.65 increased likelihood of adequate consults. Women who participated in medical examination before pregnancy were 63% less odds of having sufficient antenatal care. Women being attended to by midwives and nurses were more likely to have adequate antenatal care than those seen by doctors.

The following parameters were not associated with differences in adequacy of antenatal care: educational attainment, monthly household income, geographic location, number of miscarriages, existing medical conditions, place of birth of most recent pregnancy, distance to the nearest facility, and awareness of policies (Table 5).

**Table 5:** Logistic regression analysis of factors affecting adequacy of antenatal care

	Adequate	Inadequate	OR (95%)	*p-value*
Highest educational attainment				
Grade school	3 (25.00%)	9 (75.00%)	Ref	
High school, vocational, college undergraduate	112 (50.45%)	110 (49.55%)	3.05 (0.81–11.58)	0.101
College and postgraduate course	32 (38.55%)	51 (61.45%)	1.88 (0.47–7.48)	0.369
Marital status				
Single	39 (36.79%)	67 (63.21%)	Ref	
Married	53 (50.48%)	52 (49.52%)	1.75 (1.01–3.03)	**0.046**
Common-law partner	55 (52.38%)	50 (47.62%)	1.89 (1.09–3.28)	**0.023**
Divorced/Separated*	0	1 (100%)	-	-
Employment status (employed)	27 (29.67%)	64 (70.33%)	0.37 (0.22–0.63)	**< 0.001**
Monthly household income				
Less than 20,000	130 (44.98%)	159 (55.02%)	Ref	
20,000 to more than 50,000	17 (60.71%)	11 (39.29%)	1.89 (0.86–4.18)	0.116
Geographic location (Region)				
NCR	76 (49.67%)	77 (50.33%)	Ref	
Luzon	59 (46.09%)	69 (53.91%)	0.87 (0.54–1.39)	0.550
Visayas	7 (30.43%)	16 (69.57%)	0.44 (0.17–1.14)	0.091
Mindanao	5 (38.46%)	8 (61.54%)	0.63 (0.20–2.02)	0.441
Number of past pregnancies				
1	55 (37.16%)	93 (62.84%)	Ref	
2–3	67(51.54%)	63 (48.46%)	1.80 (1.11–2.90)	**0.016**
4 or more	25 (64.10%)	14 (35.90%)	3.02 (1.45–6.29)	**0.003**
Number of abortions				
None	122 (45.19%)	148 (54.81%)	Ref	
1	19 (50.00%)	19 (50.00%)	1.21 (0.61–2.39)	0.577
2 or more	6 (66.67%)	3 (33.33%)	2.42 (0.59–9.90)	0.217
Number of prior live births				
1	62 (38.99%)	97 (61.01%)	Ref	
2–3	63 (51.64%)	59 (48.36%)	1.67 (1.04–2.69)	**0.035**
4 or more	22 (61.11%)	14 (38.89%)	2.46 (1.17–5.16)	**0.017**
Number of living children				
1	62 (38.99%)	97 (61.01%)	Ref	
2–3	68 (52.71%)	61 (47.29%)	1.74 (1.09–2.79)	**0.020**
4 or more	17 (58.62%)	12 (42.38%)	2.22 (0.99–4.96)	0.053
Existing medical illness (with)	12 (60.00%)	8 (40.00%)	1.80 (0.71–4.53)	0.212
History of pregnancy complications (with)	24 (68.57%)	11 (31.43%)	2.82 (1.33–5.97)	**0.007**
Place of birth of most recent pregnancy				
Hospital	91 (51.12%)	87 (48.88%)	Ref	
Lying-in clinic	53 (40.77%)	77 (59.23%)	0.66 (0.42–1.04)	0.073
Home	3 (33.33%)	6 (66.67%)	0.48 (0.12–1.97)	0.307
Distance to the nearest facility for antenatal care				
Less than 30 min	96 (49.48%)	98 (50.52%)	Ref	
30 min to 1 h	37 (40.66%)	54 (59.34%)	0.70 (0.42–1.16)	0.165
More than 1 h	14 (43.75%)	18 (56.25%)	0.79 (0.37–1.69)	0.548
Mode of transportation				
On foot/bike	18 (36.00%)	32 (64.00%)	0.60 (0.32–1.12)	0.111
Motorcycle	26 (50.00%)	26 (50.00%)	1.19 (0.66–2.16)	0.566
Private vehicle	15 (68.18%)	7 (31.82%)	2.65 (1.05–6.68)	**0.039**
Public transportation	108 (45.96%)	127 (54.04%)	0.94 (0.57–1.55)	0.802
Waiting time experienced				
< 15 min	14 (29.17%)	34 (70.83%)	Ref	
15–30 min	79 (47.88%)	86 (52.12%)	2.23 (1.12–4.46)	**0.023**
31–60 min	25 (45.45%)	30 (54.55%)	2.02 (0.89–4.59)	0.091
> 60 min	29 (59.18%)	20 (40.82%)	3.52 (1.51–8.19)	**0.003**
Awareness of policies (aware)	120 (45.28%)	145 (54.72%)	0.77 (0.42–1.39)	0.381
Participated in medical examination before pregnancy (with)	75 (37.31%)	126 (62.69%)	0.36 (0.23–0.58)	**< 0.001**
Healthcare provider				
Doctor	80 (50.96%)	77 (49.04%)	1.44 (0.93–2.25)	0.106
Midwife	96 (60.00%)	64 (40.00%)	3.12 (1.97–4.94)	**< 0.001**
Nurse	26 (29.21%)	63 (70.79%)	0.36 (0.22–0.62)	**< 0.001**
Traditional Birth Attendant	2 (18.18%)	9 (81.82%)	0.25 (0.05–1.16)	0.077

*Analysis for the variable was limited by the inadequate number of the characteristic category. Cells with a frequency of less than five were merged with other cells to ensure adequacy for analysis

## Discussion

The study used the best practice recommendations jointly released by the Philippine Obstetrical and Gynecological Society and the Philippine Society of Maternal-Fetal Medicine [3]. Standard timing of outpatient department visits was suggested to be at 11–13 weeks, 20 weeks, 28 weeks, 32 weeks, 36 weeks, and 37 weeks of gestation. Interim visits at 16, 24, and 34 weeks were encouraged to be scheduled via telemedicine at the provider’s discretion. No interim guidelines were released by the Philippine Department of Health pertaining to the number of antenatal care visits during the COVID-19 pandemic. Published pre-pandemic data on antenatal care coverage by the Philippine Statistics Authority (PSA) [7] included having at least four visits. This was in line with the 2016 World Health Organization recommendation of having a minimum of four antenatal visits. This recommendation, however, has already been revised to a minimum of eight contacts including one contact in the first trimester, two contacts in the second trimester, and five contacts in the third trimester. The revision was due to the evidence that perinatal deaths increase with only four antenatal visits [8].

Initiation of antenatal care during the first trimester allows timely detection and prevention of complications. Patients receive earlier guidance on nutrition, immunization, and monitoring for danger signs. Despite previous findings by Landrian et al. that women were more likely to delay initiation of antenatal care during the pandemic, our results showed that majority of the respondents (71.38%) had their first antenatal care during the first trimester as recommended. This was similar to reported pre-pandemic data by the PSA that 71% initiated antenatal visits in the first trimester. Meanwhile, it was higher than pre-pandemic reported data by Hiroguchi and Nakazawa [9] of only 63.4% of Filipino women beginning antenatal care within the first trimester. It is possible that concerns regarding the potential risk of COVID-19 infection during their pregnancy served as motivation to seek earlier care.

Despite the physical and financial barriers to seeking antenatal care, most Filipino women were still able to seek consults for their pregnancies. Majority were seen primarily via face-to-face consults. However, less than half of the respondents were able to have at least six in-person antenatal visits. Of the 318 respondents, 46.37% had six or more face-to-face antenatal visits. This is higher compared to the utilization in Ethiopia of 29.3% [5] and lower compared to India of 75% [10]. The differences may be attributed to cultural differences, variations in sociodemographic profiles, and the national health structures. In the 2017 National Demographic and Health Survey (NDHS) published by the PSA, the percentage of women with at least four antenatal care visits was 87% in 2017. In our study, the proportion of women with at least four antenatal care visits was 64.78%. This was lower than the pre-pandemic coverage. Low utilization of services may be due to movement restrictions, fear of infection, economic pressure, and disruptions to healthcare systems.

Hospital outpatient services were closed in the early phases of the pandemic due to the lockdown restrictions. A strategy employed by most countries during the pandemic was remote care via telemedicine. However, telemedicine seems underutilized for antenatal visits in the Philippines. Half of the respondents did not have any consults by this method, which may be attributed to lack of knowledge of available services, lack of internet access, and limited availability of mobile electronic devices. These services should be promoted to improve antenatal care attendance for women with low-risk pregnancies. Expanding public health initiatives to ensure access to telemedicine should be prioritized, particularly for women of lower socioeconomic status.

The respondents utilized different facilities, with most having consultations in at least two types of facilities. Community or barangay health centers provided care to 48.86% of the women. Midwives at these centers provided most of their antenatal care. Our results showed that women attended to by midwives and nurses were more likely to have more visits than those seen by doctors. Barangay health centers and midwives were more accessible to most patients. It is plausible that some women deferred having consultations at hospitals for safety concerns.

The Maternal, Newborn, Child Health and Nutrition (MNCHN) strategy was implemented to reduce maternal and neonatal deaths aimed at the community level. This entails population-wide provision of MNCHN services to any locality in the Philippines. The strategy seeks to ensure that all pregnancies are adequately managed, and all deliveries are facility-based and managed by skilled birth attendants or health professionals. Key strategies include providing universal access and utilization of services, establishing a service delivery network, organized use of instruments for health systems development, and rapid build-up of institutional capacities. The service delivery teams in the MNCHN strategy include one women’s health team per barangay and one midwife per barangay health station. Barangay-based women’s health teams should be competent in pregnancy tracking, assisting pregnant women in birth planning, reporting maternal deaths, and organizing outreach activities as necessary [11].

The proportion of pregnant women receiving antenatal care from skilled providers increased from 85% to 1993 to 94% in 2017. The various geographic regions had antenatal care coverage by a skilled provider in 91.7–98.8%, except for the Autonomous Region in Muslim Mindanao with only 68.6%. The lack of adequate representation from the different regions in our study precludes comparison. In the 2017 NDHS, midwives were the primary providers for up to 50% of women, followed by doctors (39%) and nurses (4%). This was similar to the findings in the study, where midwives attended to 50.31% of the respondents during the pandemic. This underscores the indispensable role of primary-level health care through midwives in the country’s provision of maternal care services. Midwives should be given continuous training to strengthen their capacity as community workers.

Among the sociodemographic characteristics, marital status showed a significant difference in the adequacy of antenatal care of women. Married women and those with common-law partners were 1.75 and 1.89 times more likely to get adequate consults than single women. Spousal support was related to positive effects on mothers’ mental health and overall wellbeing. The woman’s financial capability to seek antenatal consult is augmented by having support from her husband or partner. The physical, emotional, psychological, and financial support given to women improves their health-seeking behavior during pregnancy [12].

The current study did not find a significant correlation between educational status and adequacy of antenatal care. This is contrary to previous studies that demonstrate the positive association between higher education attainment and utilization of antenatal care services [13, 14]. Education improves health literacy [13]. Thus, educated women have better understanding of the benefits of antenatal care and confidence in decision-making. Aside from education, the economic status was correlated to utilization of antenatal care in earlier studies [14, 15]. A higher economic status allows women to be able to afford healthcare costs, including travel and service expenses. Meanwhile, low-income women may allocate their limited resources to the basic needs of their family. Our study should no difference on utilization based on monthly household income. This may underscore the important role of local community health centers which are more accessible to patients. These centers provide free services and may alleviate the disparity of access to antenatal care services.

Employment was negatively correlated to the adequacy of antenatal care. Previous studies have indicated that employed mothers were more likely to have adequate antenatal care [16]. In our study, however, employed mothers were less likely to have adequate antenatal care because of their less flexible schedules and salary deductions from tardiness or absences. The development of labor laws supporting maternity leaves for antenatal care should be supported. Republic Act 11,210, an act increasing the maternity leave period to 105 days, should be strictly enforced and supported by stakeholders. Likewise, mothers should be made aware of the existence of such laws.

Among the obstetric and medical characteristics, predictors of adequate antenatal care utilization are previous pregnancies, previous live births, and having living children. Women who have had previous pregnancies may have better knowledge of pregnancy-related complications, having received previous antenatal care. Likewise, they may better understand the importance of antenatal care for improving neonatal outcomes. This may also reflect past positive experiences and outcomes with antenatal visits. History of miscarriage and existing medical illnesses are thought to improve the health-seeking behavior of women in subsequent pregnancies. These women are more likely to initiate antenatal consults for guidance and care in avoiding pregnancy complications. However, these were not found to affect the adequacy of antenatal care among the study participants, Women who had complications in their previous pregnancies were more likely to have adequate antenatal care. This demonstrates their understanding of the need for stricter follow-up and monitoring schemes for women with poor obstetric histories.

Most respondents travel less than 30 min to the nearest healthcare facility reflecting the number and distribution of available facilities. This may also reflect the active participation of primary-level care at barangay health stations in providing health care. The pandemic resulted in traveling restrictions and cancellations of public transportation modalities, which affected women’s access to facilities. Women with private vehicles were 2.65 times more likely to have adequate consults than those who utilize public modes of transportation. This further underscores the disparities experienced by women of lower economic status. The national government should address issues on transportation and improve access to continue the equitable provision of services.

Once at the facilities, more than half of the respondents waited for 15 to 30 min, while 32.71% waited more than 30 min. Women who were seen at hospitals were more likely to wait more than 30 min. This is similar to the findings of Rabbani et al. [10], where women seen at hospitals had longer waiting times compared to primary health care clinics (90 vs. 30 min). Organizing the flow of patients is vital to decrease the waiting time and thus exposure of patients to possibly infected individuals. Reducing the waiting time will also improve patient satisfaction and promote a positive antenatal care experience.

The facility in which study participants delivered did not have significant correlation with the adequacy of antenatal care. Majority of the study participants sought antenatal care in community centers and lying-in clinics but ultimately delivered in hospitals. Adequate antenatal care increases the probability of utilizing skilled attendants or community health workers in developing countries [2]. The choice of facility for antenatal care and delivery was certainly affected by the pandemic. Several facilities were understaffed and may have limited their number of obstetric admissions. Referral networks with obstetricians and pediatricians were authorized to ensure non-refusal of patients within the healthcare provider network.

The study showed a positive perception of antenatal care, with all respondents agreeing on its necessity. Despite this, nearly half of the women reported cancellation of scheduled antenatal visits. Reasons for cancellations included lockdown or quarantine restrictions, transportation problems, fear of going to the hospital or contracting coronavirus, financial and employment status problems, full schedules of hospitals or clinics, and lack of companion. These were similar to an online survey among pregnant Chinese women to investigate their attitudes toward antenatal care during the pandemic [17]. About 20% of the respondents were afraid to have any in-hospital visits. More than half postponed and canceled their appointments at any point during the pregnancy because of anxiety about going to a hospital.

Periconceptional counseling is essential to improve pregnancy outcomes. Most participants have participated in counseling and medical examination before pregnancy. However, those who have participated in a medical examination before pregnancy had 63% less odds of having adequate antenatal care. There may be a need to strengthen patient education during annual gynecologic and medical examinations of reproductive-aged women to emphasize the importance of antenatal care.

The study highlighted the determinants of health behaviors and utilization of pregnant women during the pandemic. Responsive healthcare systems should recognize these indicators, create policies to address identified problems, strengthen enabling factors, and perform continuous surveillance.

## Conclusion

Despite a positive and encouraging perception of the importance of antenatal care, adequate utilization of antenatal care was hindered by access barriers. Critical drivers for antenatal care during the pandemic were lockdown restrictions, mobility restrictions, and socioeconomic status. Remote care via telemedicine services is an appealing strategy to mitigate these barriers but was not fully utilized. The pandemic highlighted the vital role of primary care facilities and healthcare workers in the augmentation of the response. Collaborative models and appropriate referral pathways should be established to include the care of vulnerable populations such as pregnant women.

### Limitations

There are several limitations of the study. Only Filipino women who self-identify as postpartum were included in the study. Due to the sampling method, the exact number of women who received the hyperlink cannot be determined. The anonymous questionnaire format is subject to self-selection bias. Characteristics of non-respondents were not obtained. Recruited women may also have a recall bias regarding the number of antenatal consults. Possible selection biases associated with an online survey distributed primarily through social media, yielding a relatively well-educated sample. The study did not evaluate the adequacy of antenatal care in terms of appropriateness in content including the procedures and processes during antenatal care.

## Data Availability

The datasets used and/or analyzed during the current study are available from the corresponding author on reasonable request.
